# Prolactin Is Associated With Insulin Resistance and Beta-Cell Dysfunction in Infertile Women With Polycystic Ovary Syndrome

**DOI:** 10.3389/fendo.2021.571229

**Published:** 2021-02-25

**Authors:** Haiyan Yang, Jie Lin, He Li, Zhangwei Liu, Xia Chen, Qianqian Chen

**Affiliations:** ^1^ Reproductive Medicine Center, The First Affiliated Hospital of Wenzhou Medical University, Wenzhou, China; ^2^ Shanghai Ji Ai Genetics and IVF Institute, Obstetrics and Gynecology Hospital, Fudan University, Shanghai, China

**Keywords:** prolactin, infertility, polycystic ovary syndrome (PCOS), insulin resistance, beta-cell function

## Abstract

**Background:**

Our study aimed to investigate if serum prolactin (PRL) levels associated with insulin resistance and beta-cell dysfunction in infertile patients with polycystic ovary syndrome (PCOS).

**Methods:**

This was a retrospective cross-sectional study performed in the reproductive medicine center of the first affiliated hospital of Wenzhou Medical University. From January 2007 to August 2018, a total of 792 PCOS and 700 non-PCOS infertile women were included. All patients’ prolactin levels were in the normal range. PCOS was diagnosed according to the Rotterdam Criteria. Anthropometric parameters, blood pressure, serum prolactin levels, sex hormones, fasting lipids, fasting plasma glucose (FPG), fasting insulin (FINS) and hepatic biological parameters were measured in all subjects.

**Results:**

Serum prolactin levels in PCOS women were significantly decreased compared with levels in non-PCOS women after adjusting for age and BMI (*P* < 0.05). Moreover, we found that prolactin levels were positively associated with high-density lipoprotein cholesterol (HDL-C) and negatively associated with age, BMI, waist circumference (WC), hip circumference (HC), luteinizing hormone/follicle stimulating hormone (LH/FSH), estradiol (E_2_), FINS, homeostasis model assessment of insulin resistance (HOMA-IR), homeostasis model assessment of β (HOMA-β), triglyceride (TG) and alanine aminotransferase (ALT) (*P* < 0.05). After adjusting for age and BMI, multiple linear regression analysis revealed that LH, LH/FSH, E_2_, FINS, HOMA-IR, and HOMA-β were negatively associated with serum PRL (*P* < 0.05).

**Conclusions:**

Low serum PRL levels within the normal range associates with a higher incidence of insulin resistance and beta-cell dysfunction in infertile women with PCOS.

## Background

Prolactin (PRL) is a multifunctional polypeptide that stimulates insulin secretion, beta-cell proliferation and survival (1–5). It is reported that circulating PRL can support islet growth *via* enhancing hepatic insulin sensitivity and the secretion of 5-hydroxytryptamine and serotonin (6). Moreover, numerous studies have documented that PRL affects metabolism homeostasis through the regulation of key enzymes and transporters related to insulin resistance, hypertension or coronary syndrome (7–12).

It is reported that the role of PRL on glucose metabolism and insulin resistance depends on its circulating concentration. In the clinic, PRL improves glucose homeostasis by increasing beta-cell mass under certain conditions such as pregnancy, whereas excessive high PRL levels in serum indicate a high-risk of obesity and dysmetabolism, such as decreased insulin sensitivity, abnormal glucose tolerance or progressive insulin resistance (13–17). It has previously been observed that high levels of PRL exacerbate insulin resistance and impair the insulin-secretory capacity in diabetic mice, in contrast to the normal adaptive increases in glucose stimulated insulin secretion through expanded beta-cell mass and insulin sensitivity realized with moderately increased PRL levels (18). Additionally, increasing evidence links low PRL levels within the normal range with markers and outcomes of metabolic dysfunction (19–21). Previous studies have shown that serum PRL levels were negatively correlated with insulin sensitivity and glucose in young individuals (22). Low PRL levels may have an adverse effect on the JAK2/STAT5 signaling pathway and depress the function of beta-cells (8, 23–27). The maintenance of high serum PRL levels within the physiological range can improve insulin sensitivity and promote the proper distribution of fat, which ultimately modifies the metabolic dysfunction (20).

Polycystic ovary syndrome (PCOS) is a prevalent endocrine and metabolic condition characterized by the disturbance of reproductive hormones, insulin resistance, abnormal glucose tolerance, hypertension and cardiovascular disease (28, 29). It has been reported that serum PRL levels were significantly decreased in patients with PCOS, possibly leading to insulin resistance and damage of beta-cells (23). Nonetheless, the function of beta-cells and status of metabolism remain unknown in infertile patients with PCOS exhibiting normal serum PRL levels.

Thus, we analyzed the association between serum PRL levels and clinical parameters, such as waist circumference (WC), hip circumference (HC), luteinizing hormone (LH), triglyceride (TG), or the homeostasis model assessment of insulin resistance (HOMA-IR), in infertile PCOS patients by a retrospective cross-sectional study, to explore the status of PRL secretion and association with insulin resistance and beta-cell function.

## Methods

### Inclusion Criteria

The study was designed as a retrospective observational study of infertile women (792 with PCOS and 700 with tubal infertility) who were initially treated by IVF-ET and referred to the reproductive center, at the First Affiliated Hospital of Wenzhou Medical University during January 2007 to August 2018. All patients’ prolactin levels were in the normal range. The detection lower limit for PRL was 20 mIU/L and if serum PRL is over 530 mIU/L, it is considered to be hyperprolactinemia (30, 31). Due to variation in detection assays and the different kit used, the normal range of PRL varies slightly among hospitals. Based on the assay and kit used at our hospital and published consensus on diagnosis and treatment of hyperprolactinemia, all patients had normal range prolactin levels below the upper reference limit (566.46 mIU/L) and above the lower reference limit (70.81 mIU/L). PCOS was diagnosed according to the Rotterdam criteria (Rotterdam, 2004). Written informed consent was obtained from all participating individuals and the study was approved by an Institutional Review Board that complies with all principles of the Declaration of Helsinki Principles Accord.

### Exclusion Criteria

Patients with hormone therapy in three months, smoking, history of ovarian function damage by radiotherapy or chemotherapy, endometriosis, adenomyosis, thyroid disorders, liver disease, kidney disease, high blood pressure, pituitary microadenoma were excluded. Patients were also excluded if she had unexplained infertility, recurrent miscarriage or previous history of adverse pregnancy, congenital abnormalities such as chromosome aberration, congenital adrenal cortex hyperplasia, Cushing’s syndrome or testosterone-secreting tumors.

### Clinical Samples

Fasting blood samples were collected between 9 to 11 am in the morning at least 2 h after wake-up and 8 h after fasting on day 2–5 of a menstrual cycle. The body height (m) and weight (kg), waist-circumference and hip-circumference were measured by experienced nurses according to standard protocols. Body mass index(BMI) was calculated as body weight in kilograms divided by body height in meters squared. Blood pressure was taken twice in an interval of 2 min after at least 10 min rest using a mercury sphygmomanometer.

### Assay

The PRL, FSH, LH, T, and E_2_ levels in blood samples were measured using chemiluminescence assay on UniCel^®^ DxI 800 Immunoassay System (Beckman Coulter, USA) with commercial kits according to manufacturer’s and supplier’s instructions. The FPG, TG, TC, LDL-C, HDL-C and hepatic function were measured by a Cobas 8000 modular analyzer kits, and FINS by a Cobas E602 automatic electrochemical luminescence analyzer according to manufacturer’s instructions.

### Calculation

1. BMI = Weight (kg)/Height (m^2^)
2. Waist-hip ratio = Waist circumference (cm)/Hip circumference (cm)
3. Waist-height ratio = Waist circumference (cm)/Height (cm)
4. HOMA-IR = Fasting blood-glucose (FPG, mmol/L) × Fasting insulin (FINS, mIU/L)/22.5
5. HOMA-β = 20 × Fasting insulin (FINS, mIU/L)/[Fasting blood-glucose (FPG, mmol/L) − 3.5] (%)
6. The normal range of prolactin levels: 70.81–566.46 mIU/L.

### Statistical Analysis

Parameters were not normally distributed and were therefore described using medians and quartiles. The rank sum test was used to compare differences between patients and controls. The correlation among variables was analyzed using the Spearman correlation analysis. Univariate logistic regression and multiple linear regression analysis were applied to reveal the association between prolactin and the index. All statistical analyses were performed using the SPSS version 22. P values < 0.05 were considered statistically significant.

## Results

### Baseline Characteristics of Study Population

Characteristics of 792 PCOS and 700 non-PCOS women are provided in **Table 1**. Prolactin, FSH, and HDL-C levels were significantly lower in PCOS compared with non-PCOS women (235.74 versus 275.13 mIU/L, 6.62 versus 7.82 IU/L, and 1.29 versus 1.40 mmol/L, respectively), whereas blood pressure, LH, LH/FSH, testosterone (T), FINS, HOMA-IR, HOMA-β, TG, TC, LDL-C, AST and ALT remained higher in PCOS versus controls after correcting for age and BMI.

**Table 1:** Clinical and biochemical data from PCOS and control patients.

Variables	PCOS (n = 792)	non-PCOS (n = 700)
Age	29(27–32.5)	31(28–35)**
Systolic pressur (mmHg)	116(106–126.5)	109(103–117)**^a^
Diastolic pressure (mmHg)	77(68–83)	72(67–77)**^a^
BMI (kg/m^2^)	23.73(21.48–26.85)	21.64(19.53–23.88)**
WC (cm)	84(77–90.5)	75(70–81)**
HC (cm)	94(90–101)	90(86–96)*
Waist–hip ratio	0.88(0.85–0.91)	0.83(0.79–0.87)**
Basal serum PRL (mIU/L)	235.74(186.85–318.03)	275.13(213.60–355.84)*^b^
Basal serum LH (IU/L)	9.09(6.26–13.44)	4.65(3.48–5.98)**^b^
Basal serum FSH (IU/L)	6.62(5.86–7.81)	7.82(6.67–9.02)**^b^
LH/FSH	1.37(0.89–1.96)	0.59(0.42–0.77)**^b^
Basal serum E_2_ (pmol/L)	180(113.5–238.5)	182.5(134.5–238)
Basal serum T (nmol/L)	2.15(1.59–2.75)	1.56(1.25–1.96)**^b^
FINS (pmol/L)	82.9(50.35–112.30)	52.75(37.6–75.6)**^a^
FPG (mmol/L)	5.2(4.9–5.5)	5.1(4.9–5.4)
HOMA-IR	2.74(1.75–3.76)	1.74(1.22–2.55)**^a^
HOMA- β	137.49(88.35–216.24)	93.45(67.08–128.42)**^a^
TG (mmol/L)	1.37(0.94–1.98)	0.93(0.69–1.24)**^b^
TC (mmol/L)	4.79(4.20–5.30)	4.48(3.99–4.98)**^a^
LDL-C (mmol/L)	2.74(2.31–3.28)	2.49(2.10–2.92)**^a^
HDL-C (mmol/L)	1.29(1.12–1.46)	1.40(1.23–1.61)**^a^
AST (U/L)	18(16–22.5)	16(14–19)**^b^
ALT (U/L)	21(15–28)	14(11–20.5)**^b^

Parameters were not normally distributed and data are presented as quartiles. The rank sum test was used to compare differences between patients and controls.

*P < 0.05 versus controls, **P < 0.001 versus controls.

a P < 0.05 versus controls after correcting for age and BMI.

b P < 0.001 versus controls after correcting for age and BMI.

### Clinical, Hormonal, and Metabolic Characteristics in Patients With Polycystic Ovary Syndrome

**Table 2** shows the clinical and biochemical characteristics of PCOS patients grouped according to the shown quartiles of prolactin levels. Patients’ prolactin levels were inversely association with age, WC, HC, basal serum LH, LH/FSH, FINS, HOMA-IR, HOMA-β, and TG, but showed a positive association with HDL-C.

**Table 2:** Clinical and biochemical data from PCOS according to quartiles of prolactin levels.

Variables	PRL ≤ 186.85 (n = 175)	186.99–235.74 (n = 170)	235.77–318.03 (n=232)	PRL>318.03 (n = 215)	P-value
Age	31(28–34)	28.5(27–31)	29(27–31)	27(26–31)**	0.000
Systolic pressure (mmHg)	121(110–128)	113(98–124)	117(106–127)	115(106–12)	0.369
Diastolic pressure (mmHg)	78(68–83)	73(62–84)	77(70–81)	78(70.5–83)	0.841
BMI (kg/m^2^)	26.13(22.83–27.89)	23.38(21.63–24.848)	22.86(20.45–25.81)	22.58(20.50–24.68)	0.095
WC (cm)	90(82–95)	82(74–88)	80(73–88)	81(78–86)**	0.000
HC (cm)	100(92–106)	92.5(88–100)	93(88–97)	93(90–98)*	0.001
Waist–hip ratio	0.89(0.85–0.92)	0.86(0.85–0.90)	0.86(0.81–0.89)	0.88(0.85–0.92)	0.110
Basal serum PRL (mIU/L)	137.11(107.06–155.76)	209.20(195.42–227.58)	275.91(265.71–287.79)	396.28(353.25–472.53)**	0.000
Basal serum LH (IU/L)	9.61(7.08–14.28)	9.83(6.21–13.94)	8.57(5.02–11.34)	9.0(6.07–12.03)*	0.001
Basal serum FSH (IU/L)	7.0(6.02–8.47)	6.45(5.30–7.16)	6.60(5.50–8.06)	6.58(6.03–7.59)	0.810
LH/FSH	1.40(1.02–1.94)	1.55(0.98–2.44)	1.21(0.76–2.03)	1.32(0.87–1.77)**	0.000
Basal serum E_2_ (pmol/L)	162(108–217)	202(125–269)	186(143–252)	182(111–218.5)	0.345
Basal serum T (nmol/L)	2.17(1.64–2.67)	2.17(1.66–2.86)	2.15(1.72–3.02)	1.91(1.35–2.69)	0.074
FINS (pmol/L)	91.6(65.3–138.1)	87.1(50.9–145.4)	72(41.3–98)	81.6(52.3–91.25)*	0.003
FPG (mmol/L)	5.2(4.9–5.5)	5.2(4.9–5.6)	5.1(4.9–5.4)	5.1(4.9–5.6)	0.372
HOMA-IR	2.81(1.3–4.19)	2.39(1.59–3.78)	2.33(1.25–3.87)	2.20(1.02–3.34)*	0.006
HOMA-β	129.77(81.75–219.72)	112.29(78.08–164.75)	111.03(67.35–193.47)	104.48(58.59–163.14)*	0.001
TG (mmol/L)	1.43(1.09–2.26)	1.32(0.93–1.88)	1.03(0.88–2.08)	1.33(0.88–1.82)*	0.017
TC (mmol/L)	4.78(4.21–5.29)	4.79(4.120–5.29)	4.74(4.06–5.35)	4.98(4.49–5.33)	0.827
LDL-C (mmol/L)	2.74(2.37–3.20)	2.45(2.10–3.26)	2.73(2.30–3.10)	2.88(2.45–3.39)	0.810
HDL-C (mmol/L)	1.23(1.05–1.33)	1.30(1.14–1.58)	1.30(1.19–1.46)	1.31(1.16–1.54)*	0.005
AST (U/L)	18(15–24)	18(17–20)	19(15–20)	19(15.5–25.5)	0.256
ALT (U/L)	21(15–30)	22(15–28)	17(13–29)	21 (15–26)	0.123

Parameters were not normally distributed and data are presented as quartiles. The rank sum test was used to compare differences between patients and controls.

*P < 0.05 versus four groups, **P < 0.001 versus four groups.

### Associations of Serum Prolactin Levels With Sexual Hormonal and Metabolic Variables in Polycystic Ovary Syndrome Patients

**Table 3** shows the analysis of bivariate associations between prolactin and hormonal and metabolic variables in patients with PCOS, which displayed a negative association between prolactin levels and age, WC, HC, Waist-hip ratio, basal serum LH, LH/FSH, E_2_, FINS, HOMA-IR, HOMA-β, TG, and ALT (*P* < 0.05 or *P* < 0.001). Additionally, we found that prolactin levels were positively associated with HDL-C (*P* < 0.05).

**Table 3:** Bivariate associations between prolactin and hormonal and metabolic variables in patients with PCOS.

Variables	R	P
Age	−0.123*	0.001
Systolic pressure (mmHg)	−0.062	0.079
Diastolic pressure (mmHg)	−0.030	0.396
BMI (kg/m^2^)	−0.086*	0.016
WC (cm)	−0.302**	0.000
HC (cm)	−0.313**	0.000
Waist–hip ratio	−0.074	0.356
Basal serum LH (IU/L)	−0.144**	0.000
Basal serum FSH (IU/L)	0.000	0.999
LH/FSH	−0.154**	0.000
Basal serum E_2_ (pmol/L)	−0.071*	0.047
Basal serum T (nmol/L)	−0.035	0.320
FINS (pmol/L)	−0.152**	0.000
FPG (mmol/L)	0.042	0.234
HOMA-IR	−0.144**	0.000
HOMA-β	−0.165**	0.000
TG (mmol/L)	−0.107*	0.004
TC (mmol/L)	−0.014	0.702
HDL-C (mmol/L)	0.084*	0.025
LDL-C (mmol/L)	−0.027	0.463
AST (U/L)	−0.048	0.173
ALT (U/L)	−0.077*	0.030

Data shown are Spearman’s rank correlation coefficients, *P < 0.05, **P < 0.001.

### Multiple Linear Regression Analysis on the Effect of Prolactin Upon Hormonal and Metabolic Outcomes in Patients With Polycystic Ovary Syndrome

**Table 4** shows serum PRL was inversely associated with waist-hip ratio, LH, LH/FSH, E_2_, FINS, HOMA-IR and HOMA-beta after excluding the influence of age and BMI in multiple linear regression analysis.

**Table 4:** Regression analysis on the effect of prolactin upon hormonal and metabolic outcomes in patients with PCOS.

Variables	PRL	Age	BMI	R	R^2^	Adjusted R^2^
WC (cm)	−0.001	2.349	2.537	0.217	0.047	0.029
HC (cm)	−0.008	−0.325*	1.867**	0.777	0.603	0.596
Basal serum LH (nmol/L)	−0.009**	−0.168*	−0.311**	0.261	0.068	0.065
LH/FSH	−0.001**	−0.025*	−0.029**	0.216	0.047	0.043
E_2_ (pmol/L)	−0.055*	1.245	−1.280	0.108	0.012	0.008
FINS (pmol/L)	−0.066*	−1.399	7.991**	0.334	0.112	0.108
HOMA-IR (log10)	−0.102*	−0.033	0.293**	0.210	0.044	0.041
HOMA-β	−0.121*	−2.877	11.709**	0.302	0.091	0.088
TG (mmol/L)	0.000	0.019	0.081**	0.319	0.102	0.098
HDL-C (mmol/L)	0.733	−0.007*	−0.031**	0.413	0.170	0.167
ALT (U/L)	−0.004	−0.046	1.819**	0.211	0.044	0.041

Multiple regression analyses were performed with metabolic and hormonal outcomes as dependent variables and prolactin, age, and BMI as explanatory variables.

Data are presented as B-values (P-levels): *P < 0.05, **P< 0.001.

### Associations of Serum Prolactin Levels With Sexual Hormonal and Metabolic Variables in Non-Polycystic Ovary Syndrome Patients

**Table 5** shows serum PRL was negative association with age, BMI and waist-hip ratio (*P* < 0.05 or *P* < 0.001) and positively correlated with basal serum LH, LH/FSH and basal serum T (*P* < 0.05) in non-PCOS patients. In multiple linear regression analysis, serum PRL was not directly correlated with waist-hip ratio, basal serum LH, LH/FSH, T, FINS, HOMA-IR and HOMA-β after adjusting for age and BMI (**Table 6**).

**Table 5:** Bivariate associations between prolactin and hormonal and metabolic variables in non-PCOS patients.

Variables	R	P
Age	−0.171**	0.000
Systolic pressure (mmHg)	−0.025	0.505
Diastolic pressure (mmHg)	0.004	0.916
BMI (kg/m^2^)	−0.130*	0.001
WC (cm)	−0.099	0.059
HC (cm)	−0.007	0.894
Waist–hip ratio	−0.142*	0.007
Basal serum LH (IU/L)	0.094*	0.013
Basal serum FSH (IU/L)	−0.040	0.292
LH/FSH	0.120*	0.001
Basal serum E_2_ (pmol/L)	0.040	0.294
Basal serum T (nmol/L)	0.076*	0.046
FINS (pmol/L)	0.018	0.018
FPG (mmol/L)	−0.012	0.752
HOMA-IR	0.012	0.755
HOMA-β	0.034	0.365
TG (mmol/L)	−0.053	0.177
TC (mmol/L)	−0.017	0.676
HDL-C (mmol/L)	0.059	0.132
LDL-C (mmol/L)	−0.021	0.587
AST (U/L)	−0.063	0.100
ALT (U/L)	−0.065	0.177

Data shown are Spearman’s rank correlation coefficients, *P < 0.05, **P < 0.001.

**Table 6:** Regression analysis on the effect of prolactin upon hormonal and metabolic outcomes in patients with non-PCOS.

Variables	PRL	Age	BMI	R	R^2^	Adjusted R^2^
Waist–hip ratio	−0.427	−0.790	1.315	0.081	0.007	−0.002
Basal serum LH (nmol/L)	−0.569	−4.056**	−4.874**	0.38	0.057	0.053
LH/FSH	−0.348	−5.951**	−5.951**	0.230	0.053	0.049
T (nmol/L)	1.157	−3.468*	1.428	0.152	0.023	0.019
FPG (mmol/L)	−0.315	1.427	4.780**	0.192	0.037	0.037
FINS (pmol/L)	1.497	−1.986	12.772**	0.439	0.439	0.189
HOMA-IR(log10)	1.319	−1.483	12.514**	0.430	0.185	0.182
HOMA-β	0.945	−2.221*	9.423**	0.343	0.118	0.114

Multiple regression analyses were performed with metabolic and hormonal outcomes as dependent variables and prolactin, age, and BMI as explanatory variables.

Data are presented as B-values (P-levels): *P < 0.05, **P< 0.001.

## Discussion

To the best of our knowledge, this is the first study to report the association between serum PRL levels within the normal range and insulin resistance and beta-cell dysfunction in infertile patients with PCOS. In the present study, we observed that serum PRL levels were correlated with insulin sensitivity and beta-cell function in infertile PCOS patients with normal PRL levels, through analysis of the association of PRL levels with WC/HC, glucose metabolism indexes, lipid metabolism indexes and sexual hormonal regulation indexes. Whereas the correlation between PRL levels and insulin sensitivity or beta-celll function was not observed in infertile non-PCOS patients with normal PRL levels. The consequences of PRL levels in PCOS patients showed a significant decline after excluding the influence of age and BMI (*P* < 0.001), compared with non-PCOS patients (exhibiting oviductal infertility).

As a clinical diagnostic standard of central obesity, WC reflects the addition of visceral and abdominal fat, which can predict obesity-related health risk and provide a key risk factor for metabolic syndrome (MS) involving the onset of insulin resistance (32–34). In addition, as an insulin resistance-related risk factor, serum PRL levels were found to have an inverse association with WC, similar to results for PCOS or hypertrichosis patients (23, 24). In addition to WC, many studies have investigated the association between HC and type 2 diabetes (T2D), cardiovascular disease, and hypertensive or dysmetabolism disorders (35–39). An epidemiologic survey of urban Tehranian women found that HC was independently and inversely associated with metabolic risk factors (40). However, recent studies have revealed that HC is an independent risk factor for MS and cardiovascular disease (41). A relatively small sample size or imprecision of the measurements cannot be excluded as possible explanations. Our findings showed that PRL levels in women with PCOS were inversely associated with WC, HC, HOMA-IR, or HOMA-β, but not with the waist-hip ratio. Hence, we deduce that low prolactin levels within the normal range may be associated with increased WC and HC and a higher risk for insulin resistance.

Circulating PRL levels exert wide effects upon glucose metabolism. Previous studies showed that high PRL disrupted glucose homeostasis and led to metabolic abnormalities (19, 42). Patients with hyperprolactinemia exhibit more insulin resistance and glucose intolerance compared with normal individuals. However, there are also increased MS- and T2D-related risks when low prolactin levels fall within the physiological range (20, 22, 27, 43–45). Our findings support an inverse association between serum PRL levels and clinical parameters including FINS, HOMA-IR and HOMA-β in women with PCOS, after adjustment of age and BMI. Furthermore, the FINS, HOMA-IR and HOMA-β in infertile women with PCOS were significant increased compared with non-PCOS women with oviductal infertility. Thus, we propose that serum PRL levels in infertile women with PCOS may be a predictor for insulin resistance and a functional deficiency of beta-cells.

In the analysis of reproductive hormones, we found that serum PRL exhibited inverse associations with LH, LH/FSH and E_2_ levels, but was not directly correlated with either T or FSH levels. Excessive PRL reduces the secretion of FSH and LH *via* suppression of gonadotrophin-releasing hormone (GnRH) synthesis and release (46–51). Therefore, we predict that higher PRL levels within the normal range may also decrease the production of gonadotrophins. Moreover, emotional changes and a reduced quality-of-life in PCOS patients may promote dopamine secretion, which may reduce PRL levels, and lead to the inverse association between prolactin and LH or LH/FSH (52, 53). Hence, we conclude that low PRL levels may increase the levels of LH and LH/FSH.

Furthermore, we found the PRL levels were also inversely associated with TG and positively associated with HDL-C. TG, TC and LDL-C were significantly higher in PCOS compared with non-PCOS women after correcting the influence of BMI. Thus, there is significant correlation between metabolic abnormalities and serum PRL. We suspect that lower prolactin levels within the normal range may lead to dyslipidemia. Additionally, the index of ALT and AST was higher in PCOS compared with non-PCOS patients (none exhibited hepatitis or liver dysfunction), after controlling for BMI, and PRL was inversely associated with ALT. Hence, low PRL levels within the normal range may have association with higher prevalence of liver damage in PCOS. A recent clinical study into the role of PRL in the development of non-alcoholic fatty liver disease (NAFLED) suggested that there was a negative association between PRL and the presence of NAFLED. Lower PRL levels were found in patients with severe hepatic steatosis compared with those displaying mild and moderate hepatic steatosis (54). Moreover, the results revealed a novel association between the central nervous system and liver, whereby PRL/PRLR improved hepatic steatosis *via* the CD36 pathway.

With regard to the research methods, some limitations need to be acknowledged. For instance, we cannot draw causality from simple correlations in a retrospective study. Prospective studies are needed to testify their correlation, and future research will allow a more detailed investigation of all parameters such as glucose tolerance testing, insulin releasing test or abdominal ultrasonography. In addition, considering that the secretion of PRL is pulsatile and follows a circadian rhythm with the highest plasma concentration reached during sleep, and the lowest observed in the morning about 2–3 h after waking up (55), several dynamic tests of PRL secretion may be needed.

## Conclusions

Our clinical study lend support to the assumption that serum PRL levels within the normal range associates with glucose metabolism changes in infertile women with PCOS, suggesting that PRL may be a sensitive marker to predict insulin resistance and dysfunction of beta-cells. Further studies are warranted to confirm this association.

## Data Availability Statement

All relevant data are contained within the article: the original contributions presented in the study are included in the article. Further inquiries can be directed to the corresponding authors.

## Ethics Statement

The studies involving human participants were reviewed and approved by The Ethics Committee of The First Affiliated Hospital of Wenzhou Medical University. The patients/participants provided their written informed consent to participate in this study.

## Author Contributions

HY contributed to the conception, data analysis, and draft writing. JL was involved in the acquisition of data. ZL was involved in the execution. HL provided suggestions on the study design. XC contributed to the conception and design of study. QC contributed to conception and study design and revised the article. All authors contributed to the article and approved the submitted version.

## Funding

This work was supported by the National Natural Science Foundation of China (No. 81901551).

## Conflict of Interest

The authors declare that the research was conducted in the absence of any commercial or financial relationships that could be construed as a potential conflict of interest.

## Abbreviations

PRL, Prolactin; PCOS, Polycystic ovary syndrome; FPG, Fasting plasma glucose; FINS, Fasting insulin; HDL-C, High-density lipoprotein cholesterol; LDL-C, Low density lipoprotein-cholesterol; WC, Waist circumference; HC, Hip circumference; LH, Luteinizing hormone; FSH, Follicle stimulating hormone; E_2_, Estradiol; HOMA-IR, Homeostasis model assessment of insulin resistance; HOMA-β, Homeostasis model assessment of β; TC, Total cholesterol TG, Triglyceride; ALT, Alanine aminotransferase; HPL, Hyperprolactinemia; MS, Metabolic syndrome; T2D, Type 2 diabetes; GnRH, Gonadotrophin-releasing hormone; NAFLED, Non-alcoholic fatty liver disease.
